# Supplementary material for: Helping Rabbits Cope with Veterinary Acts and Vaccine-Related Stress: The Effects of the Rabbit Appeasing Pheromone (RAP)
Source: Animals (Basel). 2024 Dec 9;14(23):3549. doi: 10.3390/ani14233549 (PMC11639917; doi:10.3390/ani14233549)
Supplement: Supplementary file 1 [file animals-14-03549-s001.zip › Asproni_et_al_suppl_mat/S1_Asproni_et_al.pdf]

VAS - Clinical consultation and weighting

Rabbit information

Cage: ___________ ID: ___________

Date:___________________ Time: ______________________________

Operator: ____

Instruction

Make a sign on the following scales in order to indicate your perception on rabbit's **reactivity** and **state**.

During the procedure, how do you estimate rabbit ***reactivity****:*

The rabbit is agitated, it moves (body, abrupt movements of the hind legs), it tries to escape from the restraint

The rabbit is not agitated, it is adapted to the situation, it does not perform abrupt movements, it has not sudden reactions

During the procedure, how do you estimate rabbit ***state***:

The rabbit is very tense/inhibited: in squatting position, the muscles are contracted, the ears are positioned behind or flattened on the back

The rabbit is in a relaxed position, lying down. The rabbit is confident. The ears are in vertical position.

VAS - Vaccination

Rabbit information

Cage: ___________ ID: ___________

Date:___________________ Time: ______________________________

Operator: ____

Instruction

Make a sign on the following scales in order to indicate your perception on rabbit's **reactivity** and **state**.

*During the procedure, how do you estimate rabbit **reactivity**:*

The rabbit is agitated, it moves (body, abrupt movements of the hind legs), it tries to escape from the restraint	The rabbit is not agitated, it is adapted to the situation, it does not perform abrupt movements, it has not sudden reactions

*During the procedure, how do you estimate rabbit **state**:*

The rabbit is very tense/inhibited: in squatting position, the muscles are contracted, the ears are positioned behind or flattened on the back	The rabbit is in a relaxed position, lying down. The rabbit is confident. The ears are in vertical position.
